# Comparison of different methods for post-therapeutic dosimetry in [^177^Lu]Lu-PSMA-617 radioligand therapy

**DOI:** 10.1186/s40658-021-00385-4

**Published:** 2021-05-05

**Authors:** Florian Rosar, Niklas Schön, Hendrik Bohnenberger, Mark Bartholomä, Tobias Stemler, Stephan Maus, Fadi Khreish, Samer Ezziddin, Andrea Schaefer-Schuler

**Affiliations:** Department of Nuclear Medicine, Saarland University–Medical Center, Kirrberger Str. Geb. 50, D-66421 Homburg, Germany

**Keywords:** PSMA, Dosimetry, Radioligand therapy, Hybrid, ^177^Lu, Prostate cancer

## Abstract

**Background:**

Dosimetry is of high importance for optimization of patient-individual PSMA-targeted radioligand therapy (PSMA-RLT). The aim of our study was to evaluate and compare the feasibility of different approaches of image-based absorbed dose estimation in terms of accuracy and effort in clinical routine.

**Methods:**

Whole-body planar images and SPECT/CT images were acquired from 24 patients and 65 cycles at 24h, 48h, and ≥96h after administration of a mean activity of 6.4 GBq [^177^Lu]Lu-PSMA-617 (range 3–10.9 GBq). Dosimetry was performed by use of the following approaches: 2D planar-based dosimetry, 3D SPECT/CT-based dosimetry, and hybrid dosimetry combining 2D and 3D data. Absorbed doses were calculated according to IDAC 2.1 for the kidneys, the liver, the salivary glands, and bone metastases.

**Results:**

Mean absorbed doses estimated by 3D dosimetry (the reference method) were 0.54 ± 0.28 Gy/GBq for the kidneys, 0.10 ± 0.05 Gy/GBq for the liver, 0.81 ± 0.34 Gy/GBq for the parotid gland, 0.72 ± 0.39 Gy/GBq for the submandibular gland, and 1.68 ± 1.32 Gy/GBq for bone metastases. Absorbed doses of normal organs estimated by hybrid dosimetry showed small, non-significant differences (median up to 4.0%) to the results of 3D dosimetry. Using 2D dosimetry, in contrast, significant differences (median up to 10.9%) were observed. Regarding bone metastases, small, but significant differences (median up to 7.0%) of absorbed dose were found for both, 2D dosimetry and hybrid dosimetry. Bland-Altman analysis revealed high agreement between hybrid dosimetry and 3D dosimetry for normal organs and bone metastases, but substantial differences between 2D dosimetry and 3D dosimetry.

**Conclusion:**

Hybrid dosimetry provides high accuracy in estimation of absorbed dose in comparison to 3D dosimetry for all important organs and is therefore feasible for use in individualized PSMA-RLT.

## Introduction

Prostate carcinoma is one of the most malignant diseases in men [1]. A significant number of patients with prostate carcinoma ultimately progresses to the metastatic castration-resistant stage of prostate carcinoma (mCRPC) [2, 3]. In this stage, radioligand therapy targeting the prostate-specific membrane antigen (PSMA) is a promising therapy option, especially in patients who progressed after first/second line therapies as chemotherapy (docetaxel, cabazitaxel) and next-generation androgen receptor signaling inhibitors (abiraterone, enzalutamide) [4–7]. PSMA-targeted radioligand therapy (PSMA-RLT) with [^177^Lu]Lu-PSMA-617 showed encouraging results in several retrospective studies [8–11] and in a recent prospective study [12]. However, there is physiological expression of PSMA in normal organs such as the kidneys, the liver, and the salivary glands [13–15], resulting in undesired irradiation of these organs. This may cause radiotoxicity in the kidneys and salivary glands, probably leading to renal failure and xerostomia, respectively, and thus limiting the accumulative therapy doses and the number of therapy cycles. Both organs are therefore classified as organs at risk (OaR) for PSMA-RLT. Due to the physical properties of ^177^Lu (10.4% emission of 208 keV photons), post-therapy scintigraphy imaging is possible, allowing the estimation of absorbed doses to normal organs and tumor lesions [16]. Different 2D and 3D approaches for post-therapy dosimetry are known [17–21]. While 3D-based methods obviously offer the most valid data, performing multiple SPECT/CT scans is a tremendous task for both, patients and facilities regarding logistics and resources. Dosimetry methods based on 2D planar scintigraphy on the other hand seem to be less time-consuming and therefore mostly applied, but respective results are less accurate [22]. Recently, hybrid dosimetry combining planar and SPECT/CT imaging was proposed as a valuable approach in dosimetry of peptide receptor radionuclide therapy (PRRT) of neuroendocrine tumors. This method offers a simplification in terms of image acquisition but maintaining the required accuracy in calculated absorbed dose results [23]. However, a detailed evaluation of this hybrid method on data of mCRPC patients including estimation of absorbed doses to normal organs and tumor lesions is still missing.

Therefore, the aim of the present study was to compare absorbed dose values determined with altogether three dosimetry approaches, 2D planar-based dosimetry, 3D SPECT/CT-based dosimetry, and hybrid dosimetry combining planar scintigraphy and SPECT/CT, and to evaluate whether the accuracy of the hybrid method is suitable for use in clinical routine.

## Methods

### Patients and ethics

In this retrospective study, dosimetry data obtained from *N* = 24 mCRPC patients were analyzed. All patients received multiple pretreatments including chemotherapy and hormonal therapy (androgen deprivation therapy, enzalutamide, abiraterone) before receiving PSMA-RLT. Patient characteristics are summarized in Table 1. [^177^Lu]Lu-PSMA-617 was synthesized and administered according to EANM guidelines [24]. In order to prevent side effects, each patient received intravenous hydration (0.9% NaCl) 30 min prior to administration of the radioligand until 120 min post-injection and an external cooling of the salivary glands using cooling pads. PSMA-RLT was performed on a compassionate use basis under the German Pharmaceutical Act §13 (2b). Patients gave written consent after being thoroughly informed about the risks and potential side effects of this intervention. Additionally, patients consented to publication of any resulting data in accordance with the Declaration of Helsinki. Retrospective analysis approval was waived by the local institutional review board.

**Table 1:** Patient characteristics

Characteristics	*n* (%)/mean [min–max]
Age	71 [61–88]
Pretherapeutic PSA value [ng/ml]	591 [14–3277]
Pretherapeutic ECOG PS^a^	
0	7 (29.2%)
1	16 (66.7%)
2	1 (4.2%)
**Previous treatments**	
Prostatectomy	11 (45.8%)
Radiation	15 (62.5%)
Androgen deprivation therapy	24 (100%)
Enzalutamide or abiraterone	23 (95.8%)
Enzalutamide	20 (83.3%)
Abiraterone	22 (91.7%)
Enzalutamide and abiraterone	19 (79.2%)
Chemotherapy	19 (79.2%)
Docetaxel	19 (79.2%)
Cabazitaxel	8 (33.3%)
Docetaxel and cabazitaxel	8 (33.3%)
^223^Ra therapy	8 (33.3%)
^153^Sm therapy	1 (4.2%)
**Sites of metastases**	
Bone	24 (100%)
Lymph node	18 (75%)
Other	10 (41.7%)
**Tumor load according to** [25]	
High	11 (45.8%)
Medium	13 (54.2%)
Low	0 (0 %)

^a^Eastern Cooperative Oncology Group Performance Status

### Image acquisition

Image acquisition was performed on a Philips BrightView XCT (Philips Medical Systems, Hamburg, Germany) hybrid scanner equipped with medium energy parallel-hole collimators. Planar whole-body images as well as serial SPECT/CT scans (either of the head and neck or from the liver down to the pelvis) were acquired on days 1, 2, and 4 post-injection (approx. 24h, 48h, and ≥96 h p.i.). For reasons of practicability and in order to minimize stress for the patients, either an abdomen SPECT/CT or a head and neck SPECT/CT was performed. Patients were then assigned in two groups of approximately equal size. SPECT/CT over the head and neck region was acquired preferentially in patients with suspected bone lesions in this region. In total, 13/24 patients received whole body scintigraphy and abdomen SPECT/CT for dosimetry of the kidneys and liver and 11/24 patients received whole body scintigraphy and head and neck SPECT/CT for dosimetry of salivary glands. The energy window was set to 208 keV with a width of 20% as proposed in previous studies [21, 22]. A respective low scatter window (187 keV, 15%) was defined in order to perform scatter correction on the whole-body images according to the dual-energy window technique proposed by MIRD (*MIRD Pamphlet No. 16* [17]). For whole body acquisition, the scanning speed was 15 cm/min on day 1 and day 2 and 12 cm/min on day 4. The matrix size was 256 × 1024, and the pixel size 4.66 × 4.66 mm^2^. The day before radiopharmaceutical administration, a whole-body blank scan and a whole-body transmission scan of the patient using a flat phantom filled with aqueous solution of ^177^Lu were acquired. The corresponding images were used to correct the planar whole-body data for attenuation.

SPECT projections were acquired using 60 projections over 360° and a frame-time duration of 25 s. CT images were acquired in low-dose technique using an X-ray tube voltage of 120 keV and a tube current of 10 mA. The matrix and the pixel size were 256 × 256 and 2.33 × 2.33 mm^2^, respectively. CT data was used to calculate an attenuation map at 100 keV, which was converted to the respective emission energy of 208 keV of ^177^Lu and applied for attenuation and scatter correction of SPECT data. The respective procedure was implemented by the manufacturer and is described in detail in [26]. Iterative SPECT image reconstruction parameters were chosen to be in accordance with the specifications proposed by the MIRD pamphlets 23 and 26 [18, 19]. An iterative 3D-ordered subset expectation maximization (OSEM) algorithm was used employing 3 iterations, 8 subsets, Butterworth filtering (0.5, 10th order), and a slice thickness of 4.66 mm. In order to prevent edge and oscillation artifacts, methods of resolution recovery had not been included in the reconstruction. CT images were converted to a matrix and pixel size of 128 × 128 and 4.66 × 4.66 mm^2^ and fused with the SPECT slices.

### Calibration and recovery correction

Calibration factors for planar scintigraphy and SPECT were determined according to the specifications and requirements of the dosimetry software package applied. For planar whole-body scintigraphy, a vial filled with approx. 50 ml of ^177^Lu solution with known activity was measured during each whole-body scan of the patient. After co-registration of the planar whole body scintigrams, the count rate in a region-of-interest (ROI) surrounding the vial was determined and used to calculate the actual gamma camera response for ^177^Lu in counts/MBq. For SPECT acquisition, the camera calibration factor was determined following one of the approaches proposed by MIRD (*MIRD Pamphlet No. 26* [18]). To convert the measured voxel values in the reconstructed SPECT images to ^177^Lu activity, a large water cylinder (6595 ml) containing a well-calibrated source of ^177^Lu was scanned applying the same acquisition protocol and reconstruction method as used in the patient studies (including all corrections). From this measurement, the calibration factor was determined in units of Bq/cps. The respective activity values were then corrected applying the ^177^Lu recovery coefficients (RC) for the reconstruction setting used depending on the estimated object volume. The RCs were determined by phantom measurements on both a NEMA/IEC standard phantom and a cylindrical phantom enclosing fillable glass spheres of different volumes (3 up to 170 ml) [27]. With the applied reconstruction setting, a RC of 0.85 was reached for volumes ≥ 20 ml, which decreased to values of 0.79, 0.67, and 0.59 for sphere volumes of approximately 13 ml, 10 ml, and 6 ml, respectively. For RC correction, sphere volumes were used matching the organ or tumor volumes determined by SPECT/CT. Contrast-recovery was not considered in this study as high values of signal-to-background ratio were observed for all included tissues (≥7 for the liver, >100 for all other organs/metastases).

### Dosimetry calculation

In this study, dosimetry calculations were performed for the following tissues: kidneys, liver, salivary glands (parotid gland and submandibular gland), and bone metastases. Results of the following three different dosimetry methods were compared:

1. Planar-based dosimetry with three whole-body acquisitions (in the following referred to as “2D method”),
2. SPECT/CT-based dosimetry with three SPECT/CT acquisitions over the abdomen or head and neck, respectively (in the following referred to as “3D method”),
3. Hybrid dosimetry based on three planar whole body scintigrams and one SPECT/CT, which was used to calibrate the respective time-activity curve (in the following referred to as “hybrid method”).

**Fig. 1:**
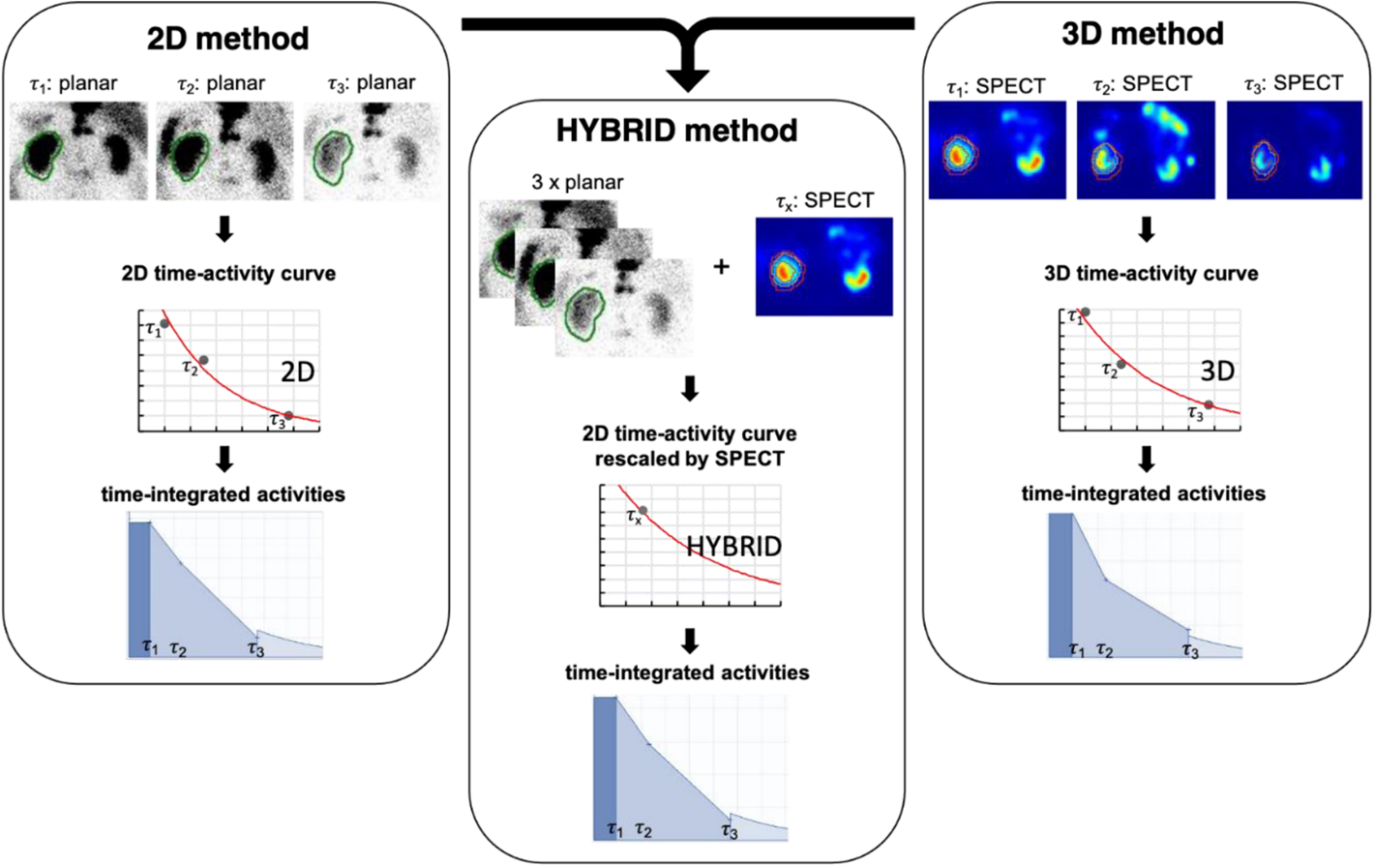
Schematic dosimetry workflow for the three different methods exemplarily shown for the data of the kidney. Absorbed dose estimation was performed by use of the software platform QDOSE

Absorbed dose calculation was performed using the QDOSE program package (ABX-CRO, Dresden, Germany). In this package, different workflows for dosimetry can be composed. The workflow we used for three dosimetry methods is described in detail in the following section and schematically depicted in Fig. 1. Estimation of absorbed dose was performed by the implemented IDAC 2.1 software [28–30] applying the IDAC reference man for the kidneys and the liver and the sphere model for the salivary glands and bone metastases, respectively. For all dosimetry methods, patient-specific organ mass adjustment is available in QDOSE. Organ masses were determined using the volume of each organ delineated from the CT images (PACS software, SECTRA, Linköping, Sweden) and the respective biological tissue density [31]. For this purpose, diagnostic CT images had been used which were acquired as part of the diagnostic workup before therapy.

#### 2D method

For 2D dosimetry, the conjugate-view method (*MIRD pamphlet No. 16* [17]) was used to calculate the organ activities from the anterior-posterior planar images. As a first step, co-registration of the planar images was performed. Boundary ROI enclosing the organs as well as background ROI near the respective organs or bone metastases was drawn in the whole-body images of the three time points. Solely bone metastases without overlap with tissues of high uptake were chosen for analysis. For the liver and kidneys, in cases of overlapping tissues with high uptake, as, e.g., parts of the gastrointestinal tract, the organ ROI did not encompass the whole organ but solely its non-overlapping part. The respective area was approximated based on the CT volume by geometrically comparing the respective contours over the entire organ and the non-overlapping fraction. The number of counts of the whole organ was then extrapolated to the entire volume assuming homogeneous uptake within the organ. In cases where one kidney was substantially overlapped by extra-renal radioactivity uptake, only the contralateral kidney was used. Attenuation correction was accomplished as proposed by MIRD (*MIRD pamphlet 16* [17]). A respective attenuation map was calculated from a blank and a patient whole body transmission scan thereby taking into account the respective thickness of the body and the organs determined by CT. Background correction was performed by determining the activity per pixel in the respective background ROI and then scaling to the number of pixels in the organ ROI. A threshold-based segmentation was applied and approved by an experienced physicist and an experienced nuclear medicine physician to delineate the organ within a manually drawn boundary ROI taking into account the anterior as well as the posterior image. The next step comprised mono-exponential regression of the serial measured activities using weighted least squares method and estimation of the time-integrated activities (TIA) and the time-integrated activity coefficient (TIAC) in the source regions by integration. As an approximation, constant activity between *t* = 0 and the first acquisition time point was assumed, and the integration method for the first-time interval was based on the trapezoidal method. Between the first and the last time point, a trapezoidal integration was used, whereas after the last time-point, mono-exponential integration was performed considering only physical decay.

#### 3D method

For 3D dosimetry, voxel values were automatically converted to activity values applying the calibration factor entered in units of Bq/cps. Volumetric co-registration of the different time points was performed by taking the first SPECT/CT scan as reference. Boundary VOI (volume-of-interest) was manually drawn in the first SPECT/CT image, which solely enclosed the source organs avoiding neighboring structures and were then copied onto the other SPECT images. Manual adjustment of the boundary VOI for each time point was possible. Volume and activity estimation were performed on each SPECT series within the boundary applying a fuzzy locally adaptive Bayesian (FLAB) segmentation algorithm for automatic volume delineation [32]. The respective results were then corrected applying the volume-dependent ^177^Lu recovery coefficients. The TIA and the TIAC in source regions were calculated as described for the 2D method above.

#### Hybrid method

For hybrid dosimetry, the 2D kinetics in the source regions derived by planar scintigraphy were combined with the respective activity estimation of the SPECT on day 1 or day 2. Here, the activity estimation of the respective SPECT was used to rescale the 2D-derived time-activity curve (*MIRD pamphlet No. 16* [17] and *No. 23* [19]).

For all three methods, the TIA and the TIAC were converted to absorbed dose estimates using the IDAC-Dose 2.1 reference man or the sphere model, respectively.

### Data analysis and statistics

All continuous data are expressed as the mean, median, and standard deviation (SD). The data were analyzed for difference in absorbed dose to all organs and to bone metastases using a Wilcoxon-Mann-Whitney test. In addition, agreement between the three dosimetry methods in terms of systematic and random differences in estimated absorbed dose in Gy/GBq [^177^Lu]Lu-PSMA-617 was investigated using Bland-Altman analysis [33]. The relative difference in absorbed dose between any two of the three methods was compared to the mean dose of the two methods. Results of Bland-Altman were summarized quantitatively in terms of the limits of agreement (LoA) being defined as the mean difference ± 1.96 standard deviations (mean diff ±1.96*SD) [33]. All analyses were performed using SPSS (version 26, SPSS Inc, Chicago, USA) and GraphPad (version 8, GraphPad Software, San Diego, USA); *p* values of less than 0.05 were considered statistically significant.

## Results

### Therapy details

In this study, a total of *n* = 65 cycles of [^177^Lu]Lu-PSMA-617 RLT of *N* = 24 patients were evaluated. On average, 3 cycles (range 1–6) per patient were analyzed. The mean administered activity of [^177^Lu]Lu-PSMA-617 was 6.4 GBq per cycle (range 3–10.9 GBq).

### Dosimetry results

19/65 dosimetry data sets were acquired for the head and neck region and 46/65 for the abdominal region. In total, investigation involved *n* = 38 parotid glands, *n* = 38 submandibular glands, *n* = 46 kidney pairs, *n* = 43 livers (3 were excluded due to multiple liver metastases), and *n* = 83 bone metastases. Dosimetry estimates in parotid and submandibular glands, the kidneys, the liver, and in bone metastases for the three methods are summarized in Table 2. Consistent results were observed for all three dosimetry methods: the liver received the lowest, whereas the parotid gland received the highest absorbed doses among the normal tissues. Regarding the bone metastases, the absorbed dose was on average two-fold higher than that of the parotid gland for all methods. Comparing the results of individual patients and cycles, large differences in absorbed dose were observed resulting in standard deviations up to 84%, most pronounced for the bone metastases (Table 2). Table 3 presents median of differences of the absorbed dose estimated by 2D and hybrid dosimetry compared to results of the 3D reference method. Median differences between the dosimetry methods were most pronounced for the kidneys followed by the parotid gland. Using the 2D method, the doses to these organs were significantly underestimated (−10.9% and −9.8%, both *p* < 0.01), and the differences were higher than those of the hybrid method (−4.0% and 0.3%). The use of hybrid dosimetry resulted in only slight underestimation of the dose to the kidneys as well as slight overestimation of the dose to the liver and the salivary glands (Table 3). However, these differences were found to be statistically non-significant (Table 3). In contrast, significantly different absorbed doses to bone metastases were observed for both methods compared to the results of 3D dosimetry.

**Table 2:** Mean absorbed dose per administered activity [Gy/GBq] in normal tissues and bone metastases estimated by the three dosimetry methods

Mean dose [Gy/GBq]	2D	Hybrid	3D
Parotid gland	0.75 ± 0.34	0.81 ± 0.34	0.81 ± 0.34
Submandibular gland	0.71 ± 0.36	0.73 ± 0.39	0.72 ± 0.39
Kidneys	0.49 ± 0.31	0.52 ± 0.27	0.54 ± 0.28
Liver	0.09 ± 0.04	0.10 ± 0.05	0.10 ± 0.05
Bone metastases	1.42 ± 0.99	1.55 ± 1.28	1.68 ± 1.32

**Table 3:** Median differences of estimated absorbed dose in normal tissues and bone metastases between 2D and 3D method, and between hybrid and 3D method

	Median of differences 2D vs. 3D			Median of differences HYBRID vs. 3D		
	[Gy/GBq]	[%]	*p*-value	[Gy/GBq]	[%]	*p*-value
Parotid gland	−0.080	−9.8	**0.003**	0.002	0.3	0.685
Submandibular gland	−0.003	−0.4	0.950	0.014	1.9	0.440
Kidneys	−0.059	−10.9	**0.005**	−0.021	−4.0	0.060
Liver	−0.004	−3.5	0.093	0.003	2.9	0.100
Bone metastases	−0.074	−4.4	**<0.0001**	−0.117	−7.0	**<0.0001**

**Fig. 2:**
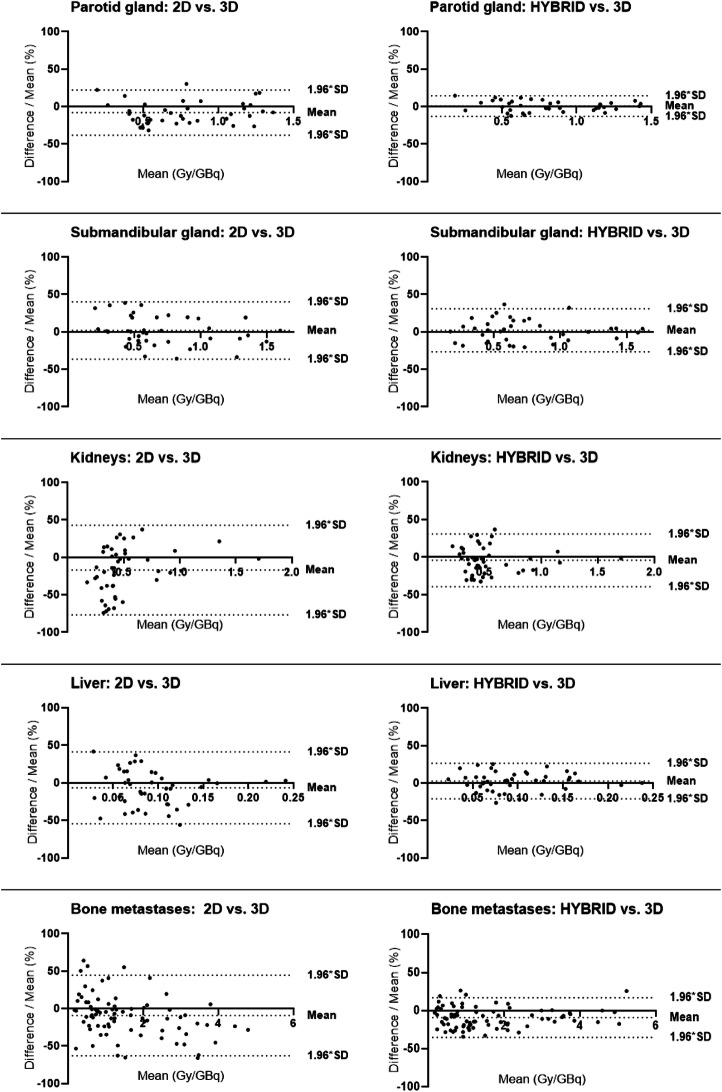
Bland-Altman plots for parotid gland, submandibular gland, kidneys, liver, and bone metastases presenting pairwise quantification of the limits of agreement (dashed lines) of the relative absorbed dose difference for 2D dosimetry and hybrid dosimetry, respectively, compared to the results of 3D dosimetry

The results of the Bland-Altman analysis of the absorbed dose per administered activity considering the alternative dosimetry methods versus the reference 3D method are provided in Fig. 2. The respective LoA for normal tissues and bone metastases between the considered method and the 3D dosimetry data as reference are shown in Table 4.

**Table 4:** Results of Bland-Altman analysis in terms of the percent average of the difference and the respective ±1.96*SD for the normal tissues and the bone metastases

	2D vs. 3D		Hybrid vs. 3D	
	Average of the difference (%)	± 1.96*SD (%)	Average of the difference (%)	± 1.96*SD (%)
Parotid gland	−8.0	30.2	+0.7	13.7
Submandibular gland	+1.9	38.2	+2.1	28.7
Kidneys	−16.9	59.8	−4.1	35.1
Liver	−6.6	47.8	+2.7	23.8
Bone metastases	−9.5	53.3	−8.9	26.1

The largest systematic difference in the mean absorbed dose was observed for the kidney using 2D dosimetry (LoA −16.9% ± 59.8%), whereas hybrid dosimetry resulted in much lower LoA of −4.1% ± 35.1%. For the other organs, 2D dosimetry resulted in random differences to 3D with LoA up to −8.0% ± 30.2%, whereas using hybrid dosimetry the LoA were lower than +2.7 ± 23.8%. For bone metastases, relatively wide LoA were found (−9.5% ± 53.3%) with 2D dosimetry compared to LoA of −8.9% ± 26.1% with hybrid dosimetry. In summary, results of hybrid dosimetry were more consistent with 3D dosimetry data yielding smaller LoA for all normal organs and metastases (Table 4).

## Discussion

In this study, we evaluated and compared three different dosimetry methods (3D method, 2D method, hybrid method) for [^177^Lu]Lu-PSMA-617 therapy on data obtained from 65 cycles of 24 mCRPC patients. Our results demonstrate that hybrid dosimetry determination provides dose estimates with high accuracy for all organs and metastases.

Assuming that 3D is the most accurate method, our evaluation showed that the hybrid method is superior to the planar-based 2D method. For the kidneys, the salivary glands, and the liver, hybrid dosimetry yielded results that are nearly equivalent with the reference 3D method (Table 3). Using 2D dosimetry, however, statistically significant underestimation was observed for doses to the kidneys (10.9%) and the parotid glands (9.8%). Regarding the bone metastases, small, but significant differences of absorbed dose were observed for both dosimetry methods.

Moreover, the Bland-Altman analyses revealed high agreement between hybrid and 3D dosimetry with LoA being low and narrow for all organs (Table 4). In contrast, high and wide LoA for all organs were observed (Table 4) when comparing 2D with 3D, indicating substantial differences between the two methods. These findings may result in non-negligible loss of accuracy using 2D dosimetry. Hence, while the hybrid method provides results for all organs that are consistent to the 3D results, the 2D method potentially underestimates corresponding doses and may therefore have an adverse clinical impact. Therefore, this method is not advised especially for dosimetry of OaR.

With respect to metastases, accurate dosimetry using 2D or hybrid methods was only possible to a limited degree as exact delineation of bone metastases in 2D planar images and respective volume determination from CT is more problematic than for normal organs. However, this issue is also of high importance as it may allow individualized lesion-based therapy planning. It should therefore be considered in further investigations.

There are several studies addressing dosimetry of RLT with [^177^Lu]Lu-PSMA-617 [34–38]. Most of them [34–36] reported on whole-body planar scintigraphy for imaging and OLINDA/EXM to estimate absorbed organ doses. In contrast to our results for 2D dosimetry, higher doses for all organs were reported in the majority of studies (e.g., kidneys 0.49 vs. 0.72–0.99 Gy/GBq [34–36]; salivary glands 0.75 vs. 0.55–1.66 Gy/GBq [34–36]). These differences may be explained by the high and unpredictable time-dependent activity uptake in overlapping normal tissues (e.g., intestine) and tumors leading to dose overestimation. This overestimation has been well described in renal 2D-dosimetry after PRRT using radiolabeled somatostatin analogs [39] and may be similarly relevant in PSMA-RLT. Furthermore, the drawing of ROIs on multiple sequential planar images is known to be subject to inter-observer variation. In the present study, these limitations of 2D dosimetry have been addressed by using small ROIs over parts of the respective organ with no overlap to other tissues assuming homogeneous activity distribution within the organ. In addition, individual background correction on each of the sequential planar images was applied to further reduce the inaccuracy. As well, the used software application allows individual organ segmentation by activity thresholding and co-registering of ROIs in the different images and may therefore reduce inter-observer variation. Nevertheless, larger uncertainty is still associated with the 2D-based dosimetry method leading to inaccuracies in estimated absorbed organ doses.

For 3D dosimetry, most of these limitations do not exist, qualifying it as the reference method for dose estimation. So far, only a few studies using 3D dosimetry in PSMA-RLT have been published [37, 38]. Comparing our findings with the literature, liver doses are in excellent agreement with those reported by the other two groups (all 0.10 Gy/GBq). Furthermore, the mean doses to the kidneys (0.54 Gy/GBq) and to the parotid glands (0.81 Gy/GBq) were lower than those reported by Delker et al. [38] (0.61 Gy/GBq (kidneys), 1.41 Gy/GBq (salivary glands)) and only slightly higher than those observed by Violet et al. [37] (0.39 Gy/GBq (kidneys), 0.58 Gy/GBq (parotid gland)). These small deviations may result from differences in patient cohorts. Whereas the latter group evaluated each first therapy cycle of *N* = 30 patients, the group of Delker et al. evaluated the first and the second cycle in *N* = 5 patients. Our study included *n* = 65 cycles of *N* = 24 patients, with the majority of patients having received multiple therapy cycles.

To the best of our knowledge, no systematic investigation of hybrid dosimetry in PSMA therapy has been reported to date. Belli et al. [40] evaluated hybrid dosimetry of the kidney in only one mCPRC patient and reported high accuracy (a difference of 1.6 %) compared to the 3D method. This is in line with our results in a larger patient cohort (median difference −4.0%, Table 3). However, hybrid dosimetry is an established method in [^177^Lu|Lu-DOTA-TATE PRRT and has been used as the reference method in a phase II trial by Sundlöv et al. [23]. These authors concluded that hybrid dosimetry is feasible and allows individualized and safe PRRT. Our data presented here indicate that hybrid dosimetry is also feasible for PSMA-RLT. Several patients received cumulated activity up to 48 GBq in up to 8 cycles of therapy, and we observed kidney doses of up to 19 Gy and parotid gland doses of up to 23 Gy. Corresponding doses are within the accepted standard limits of 15–20 Gy used in external-beam radiotherapy of the kidneys [41] and of 20–25 Gy for the salivary glands [42]. No patient in our cohort suffered from a higher-grade xerostomia (CTCAE ≥ 3), but most patients reported a mild xerostomia (CTCAE = 1), which even tend to recover over time.

Patients benefit from the dosimetry of organs at risk and tumor lesions, but the burden and the workload for the staff need also to be considered. In our clinical dosimetry protocol, each planar whole-body scintigraphy requires about 15 min and each SPECT/CT acquisition of a single region about 25 min. The accuracy of the hybrid method could be shown to be superior to the planar-based 2D method and nearly equivalent with 3D dosimetry. The additional time needed for hybrid compared to 2D dosimetry of about 25 min was short enough for patients to accept. However, as a drawback, a SPECT/CT of a single region allows only hybrid- or SPECT-based dosimetry for this particular region. In contrast, whole-body SPECT/CT would allow whole-body dosimetry, but at the expense of an extended acquisition time of about 75 min per scan. Finally, it depends on whether the patient is willing to tolerate the burden of whole-body SPECT. If not, region-based hybrid dosimetry as performed in this study might still be the compromise. Otherwise, from a clinical perspective, advanced simplification of dosimetry as known from PRRT [43, 44] may be of further interest. Here, a promising approach was proposed by Hänscheid et al. [44], who used a theoretical approach based on a single SPECT/CT (at 96 h p.i. for PRRT) and no additional whole-body planar scans for each cycle. This may motivate to perform an independent evaluation also in PSMA-targeted therapy.

In our study, we used a commercial software application (QDOSE, ABX-CRO, Dresden, Germany) in order to standardize and simplify the dosimetry procedure for use in clinical routine, while maintaining the required accuracy. This system provides some advantages. For example, tools are included to reduce the well-known issue of interobserver variation on 2D data by realizing simultaneous segmentation of the images acquired at different time-points. On SPECT data, organ contouring by automatic segmentation algorithms and predefined determination of TIA and TIAC is implemented. In addition to the reduced time needed to acquire the data for hybrid dosimetry, which is worthwhile for the convenience of the patient and the medical staff, the QDOSE software saves additional time. It includes all steps of dosimetry starting with derivation of activity images via a calibration factor and, for 3D and hybrid dosimetry, providing voxel-based absorbed dose estimation based on IDAC 2.1. No data export is needed. Nevertheless, export of TIAC to OLINDA/EXM is possible if desired. As OLINDA/EXM is probably the most established and well-known dosimetry software, a comparison of the results by IDAC 2.1 compared with OLINDA/EXM would be of interest. However, this comparison was beyond the scope of the present work.

The empirical data reported herein should be considered in the light of some limitations. The study suffers from its retrospective nature and the limited number of patients. The relatively low number of patients was a consequence of the time-consuming and patient-exhausting data acquisition needed for dosimetry. As patients in this study only underwent 3D dosimetry for one of the two regions (abdominal or head and neck) and only under the condition of feeling comfortable, an inhomogeneous number of cycles per patient was included in this study. In addition, patient comfort and management allowed imaging only at one time point before (on the same day—for acquiring the transmission scan) and three time points after administration of the [^177^Lu]Lu-PSMA-617 during hospitalization, whereas patients would have needed to return to the hospital for a fourth, later imaging procedure. For the same reason of patient comfort, the use of an additional earlier time point (e.g., 4h or 0.5h pi) which has been discussed in literature on PRRT to improve dosimetry results could not be included in the clinical protocol [45]. In this context, further studies investigating the impact of the different phases of organ pharmacokinetic on absorbed dose estimates especially for PSMA-RLT would be of interest. Moreover, dosimetry of all bone metastases or total tumor burden, respectively, was not possible. Thus, no conclusions regarding the overall therapeutic tumor dose can be drawn. Further studies, ideally in a prospective setting, with larger patient cohorts are therefore recommended to confirm and expand our findings.

## Conclusion

Our results demonstrate that absorbed dose estimation by hybrid dosimetry provides high accuracy for PSMA-RLT in comparison to the reference 3D method. In addition, it is a less demanding method with respect to clinical resources and distress for patients. Therefore, hybrid dosimetry is feasible and qualifies for routine application in individualized PSMA-RLT.

## Data Availability

The datasets used and analyzed during the current study are available from the corresponding author on reasonable request.

## Abbreviations

CTCAE: Common terminology criteria of adverse events
FLAB: Fuzzy locally adaptive Bayesian
LoA: Limits of agreement
mCRPC: Metastatic castration-resistant prostate carcinoma
OaR: Organs at risk
OSEM: Ordered subset expectation maximization
PRRT: Peptide receptor radionuclide therapy
PSMA: Prostate-specific membrane antigen
PSMA-RLT: PSMA-targeted radioligand therapy
RC: Recovery coefficient
RLT: Radioligand therapy
ROI: Region-of-interest
SD: Standard deviation
TIA: Time-integrated activity
TIAC: Time-integrated activity coefficient
VOI: Volume-of-interest
